# Tracking canopy chlorophyll fluorescence with a low-cost light emitting diode platform

**DOI:** 10.1093/aobpla/plad069

**Published:** 2023-10-18

**Authors:** Logan E G Brissette, Christopher Y S Wong, Devin P McHugh, Jessie Au, Erica L Orcutt, Marie C Klein, Troy S Magney

**Affiliations:** Department of Plant Sciences, University of California, Davis, Davis, CA 95616, USA; Department of Plant Sciences, University of California, Davis, Davis, CA 95616, USA; Department of Plant Sciences, University of California, Davis, Davis, CA 95616, USA; Department of Plant Sciences, University of California, Davis, Davis, CA 95616, USA; Department of Plant Sciences, University of California, Davis, Davis, CA 95616, USA; Department of Geography, California State University, Sacramento, Sacramento, CA 95819, USA; Department of Plant Sciences, University of California, Davis, Davis, CA 95616, USA; Department of Plant Sciences, University of California, Davis, Davis, CA 95616, USA

**Keywords:** Chlorophyll fluorescence, light-emitting diode, photosynthesis, plant ecophysiology, remote sensing

## Abstract

Chlorophyll fluorescence measured at the leaf scale through pulse amplitude modulation (PAM) has provided valuable insight into photosynthesis. At the canopy- and satellite-scale, solar-induced fluorescence (SIF) provides a method to estimate the photosynthetic activity of plants across spatiotemporal scales. However, retrieving SIF signal remotely requires instruments with high spectral resolution, making it difficult and often expensive to measure canopy-level steady-state chlorophyll fluorescence under natural sunlight. Considering this, we built a novel low-cost photodiode system that retrieves far-red chlorophyll fluorescence emission induced by a blue light emitting diode (LED) light source, for 2 h at night, above the canopy. Our objective was to determine if an active remote sensing-based night-time photodiode method could track changes in canopy-scale LED-induced chlorophyll fluorescence (LEDIF) during an imposed drought on a broadleaf evergreen shrub, *Polygala myrtifolia*. Far-red LEDIF (720–740 nm) was retrieved using low-cost photodiodes (LEDIF_photodiode_) and validated against measurements from a hyperspectral spectroradiometer (LEDIF_hyperspectral_). To link the LEDIF signal with physiological drought response, we tracked stomatal conductance (*g*_sw_) using a porometer, two leaf-level vegetation indices—photochemical reflectance index and normalized difference vegetation index—to represent xanthophyll and chlorophyll pigment dynamics, respectively, and a PAM fluorimeter to measure photochemical and non-photochemical dynamics. Our results demonstrate a similar performance between the photodiode and hyperspectral retrievals of LEDIF (*R*^2^ = 0.77). Furthermore, LEDIF_photodiode_ closely tracked drought responses associated with a decrease in photochemical quenching (*R*^2^ = 0.69), *F*_v_/*F*_m_ (*R*^2^ = 0.59) and leaf-level photochemical reflectance index (*R*^2^ = 0.59). Therefore, the low-cost LEDIF_photodiode_ approach has the potential to be a meaningful indicator of photosynthetic activity at spatial scales greater than an individual leaf and over time.

## Teaser

We built a low-cost platform (~$250 USD) designed to measure canopy fluorescence emission at night. We compared canopy light emitting diode-induced fluorescence (LEDIF) measurements with daily information on plant physiology and canopy fluorescence measured with a more expensive spectroradiometer and found a close correspondence over the course of an imposed drought on a single plant. This tool permits continuous measurements of whole plant photochemical dynamics and the potential to track plant stress prior to visible detection or provide validation measurements for remote sensing data on solar-induced fluorescence.

## Introduction

The terrestrial biosphere acts as a crucial sink for current and future atmospheric CO_2_ through the process of photosynthesis (Turner *et al.* 2006; Beer *et al.* 2010; Ryu *et al.* 2019). Although photosynthesis can be more directly measured at the leaf level, scaling estimates of photosynthesis to the canopy and ecosystem level can be labour or model intensive, expensive and require specialized equipment (Baldocchi 2003; Ryu *et al.* 2019; Sun *et al.* 2019). Fortunately, remote sensing technologies have been developed to aid in large-scale estimates of photosynthesis, allowing us to better scale gross primary productivity (GPP) globally (Sun *et al.* 2018*a*; Ryu Ryu *et al.* 2019Xiao *et al.* 2019; ). Despite the promise of satellite-based estimates of GPP, global carbon cycle uncertainty remains high ([Bibr CIT0074][Bibr CIT0080]; ). This is because satellite data remain difficult to interpret without a mechanistic understanding of when, why and to what extent reflected or re-emitted photons co-vary with changes in photosynthesis. To aid in this, having a network of remote sensing technologies at a variety of scales—leaf, tower, aircraft, satellite—linked with plant physiological measurements, can provide invaluable insight into the temporal dynamics of plant function.

Reflectance-based vegetation indices (VIs) have been used to estimate GPP from remote sensing platforms (Peng and Gitelson 2011; Huang *et al.* 2019; Lin *et al.* 2019), but performance may be limited in certain scenarios (Xue and Su 2017). One of the most well-known VIs is the normalized difference vegetation index—NDVI, which estimates vegetation ‘greenness’ and is used to infer the health and productivity of vegetation (Tucker 1979). However, NDVI may saturate in areas of dense vegetation (i.e. high leaf area index), such as croplands and forested areas (Baret and Guyot 1991; Huete *et al.* 1997; Sun *et al.* 2018*b*). Furthermore, as NDVI is a metric of green plant biomass, it may be limited in assessing photosynthetic activity in ecosystems with little structural change, such as evergreen-dominated ecosystems (Magney *et al.* 2019; Pierrat *et al.* 2022*a*, *b*). As a result, physiologically sensitive VIs may provide an advantage for monitoring vegetation function, such as the photochemical reflectance index (PRI, Gamon *et al.* 1992). PRI changes rapidly under increasing incident light as excess energy builds due to saturating photochemistry, leading to de-epoxidation of xanthophyll cycle pigments (a subgroup of carotenoids)—violaxanthin into the photoprotective antheraxanthin and zeaxanthin—to dissipate excess energy as heat (Demmig-Adams and Adams 1996; Gamon *et al.* 1997). Thus, PRI can track fluctuations in photoprotective carotenoid pigment activity, which can then be used as a proxy for daily or even seasonal changes in photosynthetic activity (Garbulsky *et al.* 2011; Pierrat *et al.* 2022a), and as an early indicator of plant stress from environmental changes such as drought (Sarlikioti Sarlikioti *et al.* 2010; Magney *et al.* 2016; Zhang *et al.* 2017).

VIs have demonstrated that remote sensing proxies can inform our understanding of changes in plant ‘greenness’ and pigments (Glenn *et al.* 2008); however, we can further probe photosynthetic activity by understanding and measuring the fate of photons upon reaching the leaf. Inside the leaf, there are three dominant competing pathways in which absorbed light energy can be quenched by the plant. Absorbed light energy can be (i) used for photochemistry (aka photosynthesis), (ii) emitted as fluorescence or (iii) dissipated as heat—non-photochemical quenching (NPQ, Murchie and Lawson 2013; Porcar-Castell *et al.* 2014). As the three pathways are in competition—meaning that an increase in one may result in a decrease in the other pathways—measuring chlorophyll fluorescence can enable inference into the dynamics of photochemistry and NPQ (Maxwell and Johnson 2000; Baker 2008; Magney *et al.* 2017, 2020; Porcar-Castell *et al.* 2021).

Chlorophyll *a* fluorescence has been actively measured at the leaf level through pulse amplitude modulation (PAM) fluorometry for decades (Baker and Oxborough 2004). The natural progression of fluorescence research, then, has been to scale leaf-level PAM measurements to the canopy-level spectral fluorescence emissions (Mohammed *et al.* 2019; Porcar-Castell *et al.* 2021). For over a decade, solar-induced fluorescence (SIF), measured from 650 to 850 nm, has been used to measure chlorophyll fluorescence passively through satellites (Frankenberg *et al.* 2011; Joiner *et al.* 2011). SIF is measured as a red-far-red emission in the Fraunhofer lines (Frankenberg *et al.* 2011; Joiner *et al.* 2011), but it has also been quantified using the oxygen bands (Meroni *et al.* 2009). Further investigation of SIF has shown a linear relationship between SIF and GPP at the satellite level (Sun *et al.* 2017, 2018a). This is promising; however, satellite retrievals of SIF under natural sunlight require expensive, highly sensitive spectrometers necessary to resolve the Fraunhofer lines (Grossmann *et al.* 2018). Moreover, SIF is highly dynamic and represents the state of a plant during a satellite overpass—driven diurnally by changes in water availability, temperature, vapour-pressure deficit or light intensity (Verma *et al.* 2017; Magney *et al.* 2019; Pierrat *et al.* 2022b). To understand these nuances, further investigation of the relationship between SIF and GPP is necessary at smaller spatial and temporal scales, and across all ecosystems (Porcar-Castell *et al.* 2014). Therefore, specifically investigating the relationship between leaf and canopy fluorescence with photosynthesis, especially under stress events like drought, may provide novel insight into the connection between physiology and remote sensing proxies.

Detecting drought is becoming increasingly important, as droughts are becoming more frequent, widespread and intense (Allen *et al.* 2010; Chiang *et al.* 2021). The impacts of drought on plants have been extensively studied through various methods, including but not limited to, measurements of changes in stomatal conductance (Wu *et al.* 2019), cavitation (Vilagrosa *et al.* 2003; Choat *et al.* 2012; Fichot *et al.* 2015) and the use of remote sensing proxies like VIs (Wagle *et al.* 2014; Rousta *et al.* 2020). SIF, with its inherent connection to the competing pathways of absorbed light, allows stress detection to happen before any visible signs in leaf coloration (Ač *et al.* 2015; Magney *et al.* 2020; Kimm *et al.* 2021). However, the nuances of how SIF changes under drought stress have not been thoroughly studied and suggest conflicting results. In some cases, an increase in SIF and/or steady-state fluorescence has been observed during drought (Amoros-Lopez *et al.* 2006; Buddenbaum Buddenbaum *et al.* 2015; [Bibr CIT0003][Bibr CIT0046]; ; 2018Zhang *et al.* 2018). While in others, a decrease in SIF during the drought has been observed (Zarco-Tejada *et al.* 2016; [Bibr CIT0070][Bibr CIT0015]; ). It is important to note that these studies were all conducted at different spatiotemporal scales, and with different instruments. However, fundamental leaf-scale studies using PAM fluorimetry (Flexas *et al.* 2002) showed that an increase in drought stress caused a series of physiological changes in the plant, including an increase in NPQ, a decrease in photochemical quenching (PQ) and a non-linear decrease in steady-state fluorescence emission; which could lead to a change in the relationship of SIF and carbon uptake. Yet, when looking into the leaf level relationship between fluorescence and photosynthesis during a short-term drought, Helm *et al.* (2020) found that chlorophyll *a* fluorescence did not reflect a strong response to drought, yet the response was strongly observed in the stomata and rate of photosynthesis. A better understanding of how SIF changes in response to stress at the leaf and canopy level is essential for interpretation of SIF data to estimate carbon fluxes across various environments.

Expanding on the fluorescence research and relationships established through PAM and SIF, LEDs have been employed at night to actively measure canopy-level chlorophyll *a* fluorescence. Romero *et al.* (2018) successfully used LEDs to measure and model canopy fluorescence and calculate reabsorption values in a controlled plant canopy environment. In a forest consisting of Scots Pine and lingonberry, a coloured (blue, red and green) LED system was installed above the canopies and illuminated the canopy for 2 h (Atherton *et al.* 2019). In this study, using a field spectrometer at night with long integration times, they measured the quantum yield of fluorescence excited by the LED lights (red, green and blue) and coined the new nocturnal method: LED-Induced chlorophyll *a* Fluorescence—LEDIF (Atherton *et al.* 2019). Romero *et al.* (2021) built an LEDIF system and implemented it in an agricultural environment. They investigated how canopy net primary productivity varied with different water treatments and bean cultivars using passive (VIs and SIF) and active (PAM and LEDIF) remote sensing to discern changes in plant health. They found that chlorophyll *a* fluorescence, whether collected passively or actively, offered insight into plant health before any visible cues. These studies are encouraging because of their ability to detect physiological changes and give warning signs of plant stress. However, a common theme across these research investigations is that measuring fluorescence at night was done using a spectrometer, which prohibits low-cost applications. Because these measurements were done at night, we propose that a lower-cost light detection method can be used because spectral resolution is not an issue.

Our night-time LED system is unlike current self-built daytime SIF measuring systems, such as PhotoSpec (Grossmann *et al.* 2018) and FluoSpec (Yang *et al.* 2015, 2018), which require thermally stable, high spectral resolution spectrometers. To address this, we developed a simple, repeatable, easy to install, low-cost alternative to measuring chlorophyll *a* fluorescence. We measured chlorophyll fluorescence at night using a blue LED light source, which offered the potential to measure a pure fluorescence signal using low-cost photodiodes (~$250 USD) in the far-red region. Therefore, our objectives were to (i) determine if we can track changes in canopy-level chlorophyll fluorescence with a new, night-time, low-cost sensor during an imposed stress event and (ii) investigate whether these changes are reflected at the leaf- and canopy-level. To accomplish this, we compared the temporal dynamics of LEDIF from our low-cost photodiode system to that of a more expensive hyperspectral instrument. We investigated the relationship between the physiological and fluorescence parameters to contextualize and understand how our platform performed against more traditional methods.

## Materials and Methods

In this study, we investigated the relationship between physiological and fluorescence parameters to inform how our novel low-cost photodiode system performed in comparison to traditional methods, particularly focussing on the changes in canopy- and leaf-scale chlorophyll *a* fluorescence, during an imposed drought. To achieve this, we used a suite of standard instruments, including a PAM fluorimeter, a porometer and a spectroradiometer to measure a range of parameters, such as LED-induced chlorophyll fluorescence (LEDIF), stomatal conductance (*g*_sw_), PQ, NPQ *F*_v_/*F*_m_ and the photochemical reflectance index (PRI), among others (Table 1).

**Table 1.**
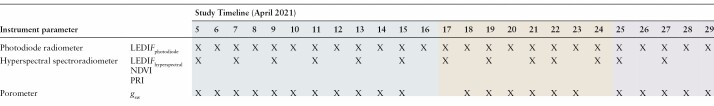
Timing of when data were taken, each instrument was used, and what data were collected. Numbers reference dates in April, 2021: that is, ‘5’ is the 5 April 2021. Blue text refers to canopy-level measurements and green text refers to leaf-level measurements. Shaded regions indicate watering period: green = pre-drought, brown = drought, purple = post-drought.

### Experimental setup

In this experimental setup, we aimed to evaluate the efficacy of our platform by conducting tests on an individual, well-established ornamental evergreen shrub, specifically the Sweet Pea Shrub (*Polygala myrtifolia*). We initiated a controlled drought and recovery experiment in early spring that began on 5 April 2021, and lasted for 25 days. Our experiment consisted of three periods: pre-drought—5 April—16 April where the plant was well-watered; drought—17 April—23 April when water was withheld; and post-drought—24 April—29 April where we began watering regularly again (Table 1). There was no precipitation during this time, and average daytime temperatures remained stable (16.5 °C ± 3 °C), with little change in daily average relative humidity (55 % ±10 %). Daily maximum temperatures ranged from 23 °C to 29 °C, and minimum from 6.5 °C to 8.5 °C, with little fluctuation over the course of the experiment. These relatively mild conditions with little variation suggest that changes in environmental conditions during the experiment were negligible compared to the imposed drought.

**Figure 1.**
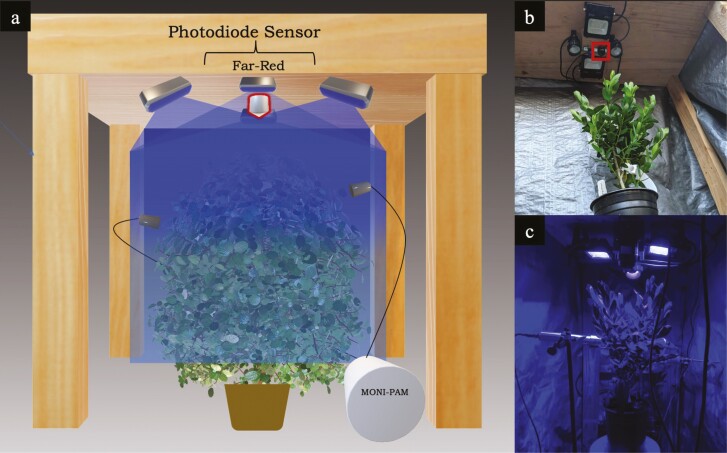
(A) Conceptual figure showing the setup and layout for novel LEDIF drought experiment. (B) View of built structure with mounted LED lights encircling the photodiode radiometer sensor (sensor outlined in red). (C) View of setup with blue LEDs on, sensors connected (photodiode: above, MONI-PAM: heads attached to leaves mid-canopy) and tarp excluding extraneous environmental lighting.

**Figure 2.**
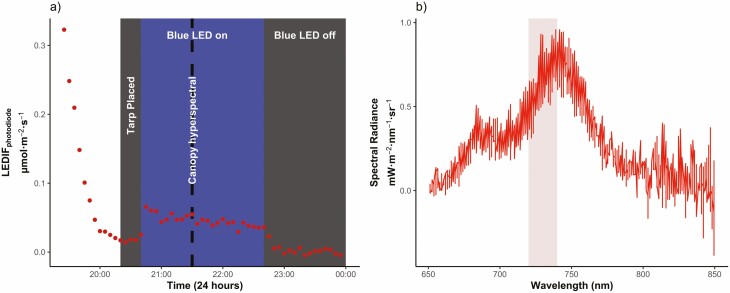
Night-time canopy measurements of *Polygala myrtifolia*. (A) The LEDIF_photodiode_ canopy-level chlorophyll *a* fluorescence immediately before, during and after the blue LED lights are turned on and off. A tarp is placed over the platform to prevent an extra light from entering the measurement area for at least 15 min prior to the LEDs being turned on. The blue LEDs are then switched on for a 2-h period. An hour after the blue LEDs are switched off, the ‘dark-period’ far-red region spectra are then taken, averaged over the following hour period. LEDIF_photodiode_ values are corrected using the averaged ‘dark-period’ values. Dashed black line indicate when the canopy hyperspectral measurements were taken. (B) displays the chlorophyll *a* fluorescence emission spectrum collected from the hyperspectral spectrometer during the ‘blue LED on’ period. The shaded region indicated the wavelengths being measured with the far-red photodiode sensor (720 nm–740 nm).

The experiment took place in a wood-built structure (Fig. 1), on an experimental plot located in northern Davis, California. Excessive shading by the structure was avoided by cutting large windows in the top panel of the structure. These windows did not interfere with the solar panels that powered the MONI-PAM, or with the ceiling-mounted photodiode radiometres and lights. Five blue LED lights (three at 5 W and two at 15 W) were mounted ~20 cm above the canopy (FZWLE RGBW 5 W LED Spot Lights; Pesken Lighting RGB + CW 15 W Flood Lights). Measured PAR values at the top of the canopy ranged from 15 to 30 µmol m^-2^ s^-1^, inducing a light level equivalent to minimal fluorescence (*F*_o_) from PAM. The lights were positioned around the photodiode sensor in a circular arrangement that enclosed but did not touch the sensor and ensured that the top of the canopy was fully illuminated. Extraneous light from surrounding plots was blocked by a heavy-duty tarp placed around the housing structure and was secured to the ground on each side from dusk until dawn, each day. After at least 15 min of being completely shaded under the tarp, the LEDs were then switched on for 2 h at night, starting no earlier than 2030 h and no later than 2130 h. The LEDs provided the irradiance necessary to actively measure night-time fluorescence in *P. myrtifolia* (Fig. 2A).

### Canopy-level measurements

#### Broadband photodiode radiometer.

A photodiode radiometer, measuring in the red/far-red regions (645 nm–665 nm, 720 nm–740 nm, respectively) with a FOV of 180°, was mounted ~20 cm from the top of the canopy (S2-131, Apogee Instruments, Inc., Logan, UT, USA). This relatively inexpensive sensor (**~**$250/sensor) provided continuous spectral measurements throughout the duration of the experiment. Data collection began on 5 April 2021. Data were logged every 5 min, throughout each day and were stored on data loggers and downloaded regularly (AT-100 microCache Bluetooth Micro Logger, Apogee Instruments, Inc., Logan, UT, USA) (Table 1). Initially, the radiometers were placed too low, causing them to pick up the direct irradiation from the adjacent blue LEDs. Therefore, the canopy-level data used for analysis began on 10 April 2021 after the sensors were adjusted (Table 1). Ultimately these data were used to quantify steady-state chlorophyll *a* fluorescence emission, in the far-red region. Data collected in the red region (645 nm to 665 nm) were not used as values were close to zero radiance, suggesting that the signal to noise was too low, despite being in the fluorescence emission spectrum that was excited by the blue LEDs (Fig. 2B).

Far-red values were summarized (daily mean and standard deviation) across the active LED light period (Fig. 2A). One hour after the LED lights were turned off, these metrics were again summarized over a 1-h period, to get the ‘dark period’ values. For example, in Fig. 2A, the tarp was placed at 2021 h, the blue LEDs were turned on at 2041 h, and then were turned off at 2241 h. For the dark period measurements, the average over 2341 h until 0041 h was taken. In this study, steady-state fluorescence values were gathered from the far-red region (720–740 nm) and will henceforth be referred to as LEDIF_photodiode_ (Fig. 2B). LEDIF_photodiode_ have also been dark corrected; mean dark period values after the LEDs were turned off were subtracted from the LED light period values (Fig. 2).

#### Canopy fluorescence with hyperspectral instrument.

LED-induced steady-state canopy fluorescence (LEDIF_hyperspectral_) was measured using a hyperspectral spectroradiometer (SVC, HR-1024i, Spectra Vista Corporation, Poughkeepsie, NY, USA). The specifications on this spectroradiometer were a 25° FOV optic fibre with an autointegration time to maximize signal, which was averaged over 1 s. The noise equivalent radiance of the instrument is < 0.8 × 10^-9^ W cm^-2^ nm^-1^ sr^-1^ at 700 nm. Measurements were taken approximately every other day during the pre- and post-drought periods, while more frequent measurements were taken during the latter half of the induced drought period (Table 1). After the lights were activated, a period of 15 min was given before measurements started, allowing the plant to adjust to the new light source and avoid the Kautsky effect (Kautsky and Hirsch 1931; Litchenthaler 1992) (Fig. 2A). Note that a tarp stayed over the structure while the night-time sampling occurred to block any extraneous light from entering. A single canopy average was obtained by repeating a series of four scans taken every 90° around the plant, with the fibre head at an angle of 45° and ~15 cm above the top of the canopy, each day that measurements were made.

These canopy fluorescence measurements were made to have a direct comparison of those collected from our new inexpensive photodiode sensor platform system. To make a more direct comparison, fluorescence values collected from the hyperspectral spectroradiometer were averaged over the same wavelengths to the broadband photodiode sensors (720 nm—740 nm). Daily canopy averages of emitted radiance in this region were then averaged over the four scans (Fig. 2B).

### Leaf-level measurements

#### Pulse amplitude modulated fluorescence.

At the leaf level, active fluorescence measurements were taken using a MONITORING-PAM device (MONI-PAM, Heinz Walz GmbH, Effeltrich, Germany). Two measuring head clips were attached to two randomly chosen leaves. Measuring light intensity was set at 1.5–22 μmol m^-2^s^-1^ at 100 Hz modulating frequency. The saturation pulse used was 8500 μmol m^-2^s^-1^ for 0.6 s.

A series of leaf-level metrics were calculated from data collected by the MONI-PAM. Initial parameters collected and descriptions are included in Table 2 (adapted from Baker 2008). The MONI-PAM took measurements continuously (24 h/day) for the duration of the experiment, but logged data every half-hour. Night-time values were parsed from data collected between 0000 h and 0500 h (i.e. *F*_m_, *F*_o_ (LED off), and ${F_{o}}^{\prime }$ (LED on)). Note that ${F_{o}}^{\prime }$ values shown in the analysis were taken when the LED was turned on at night. Daytime values were parsed from data collected between 1100 h and 1600 h (i.e. ${F_{m}}^{\prime }$, *F*_*s*_). Daily averages (across both measuring heads and day/night hours specified) were then taken to match other instrumentation for the best comparisons possible. Due to unexpected wind events, a single day was removed from one of the measuring heads.

**Table 2.** Chlorophyll fluorescence parameters collected with the MONI-PAM fluorometer, their descriptions and equations.

Parameter	Description
*F*_m_	Maximum fluorescence after saturation pulse from dark adapted leaf
${F^{\prime }}_{m}$	Maximum fluorescence under light adapted conditions
*F*_s_	Steady-state fluorescence under ambient light conditions
*F*_o_	Minimal fluorescence from dark adapted leaf
$\frac{F_{v}}{F_{m}}$	Maximum quantum efficiency of PSII photochemistry; $\frac{F_{m}-F_{o}}{F_{m}}$
${F^{\prime }}_{o}$	Minimum fluorescence under light adapted conditions
PQ	Photochemical quenching; $\frac{F_{m}}{F_{s}}-\frac{F_{m}}{{F^{\prime }}_{m}}$
NPQ	Non-photochemical quenching; $\frac{F_{m}-{F^{\prime }}_{m}\;\;\;}{{F^{\prime }}_{m}},$

#### Leaf hyperspectral reflectance.

Leaf reflectance was measured at night using the same spectroradiometer with an attachment leaf clip (HR-1024i and LC-RP Pro, Spectra Vista Corporation, Poughkeepsie, NY, USA). The sampling frequency (# of days) and timing (waiting 15 min after light activation) of measurements remained the same as the canopy-level methodology (Table 1). Leaf sampling occurred immediately after the canopy-level spectral measurements, at night during the blue LED exposure time. To obtain a complete picture of what was happening throughout the canopy, a series of 12 scans were taken: four random leaves in the top, followed by four in the middle and finally four in the bottom third of the shrub. A white panel calibration, taken on Spectralon disks that are built into the leaf clip, was taken before each ‘section’ of the shrub.

NDVI and PRI were computed for each leaf (*n* = 12). Data were summarized to get the daily plant mean and standard deviation for each index. The following equations were used to calculate NDVI and PRI, where *R* represents reflectance in the respective waveband in the subscript:


$$\begin{array}{l} \begin{array}{lll} & NDVI=\frac{R_{800}-R_{680}}{R_{800}+R_{680}}\; & PRI=\frac{R_{531}-R_{570}}{R_{531}+R_{570}}.\; \end{array} \end{array}$$

#### Porometer.

We used a LI-600 porometer to monitor stomatal conductance (*g*_sw_) (LICOR Biosciences, Lincoln, NE, USA). Default settings and auto-stabilization were used when taking measurements. Measurements were taken for 22 of the 25 days throughout the experiment (Table 1) and all samples took place within ±1 h of solar noon. The sampling structure mimicked that of the leaf-level field spectrometer measurements, where a series of 12 scans were taken: four random leaves in the top, followed by four in the middle, and finally four in the bottom third of the shrub. A daily plant average, across all 12 leaves, was then calculated.

#### Data analysis.

Data handling, processing and statistical analysis were conducted using theR programming language (R Development Core Team 2020). Linear regressions were run to compare the LEDIF_photodiode_ setup performed against more traditionally acquired fluorescence and photosynthetic performance metrics. All analysis used daily means for comparisons across multiple instrument types.

## Results

**Figure 3.**
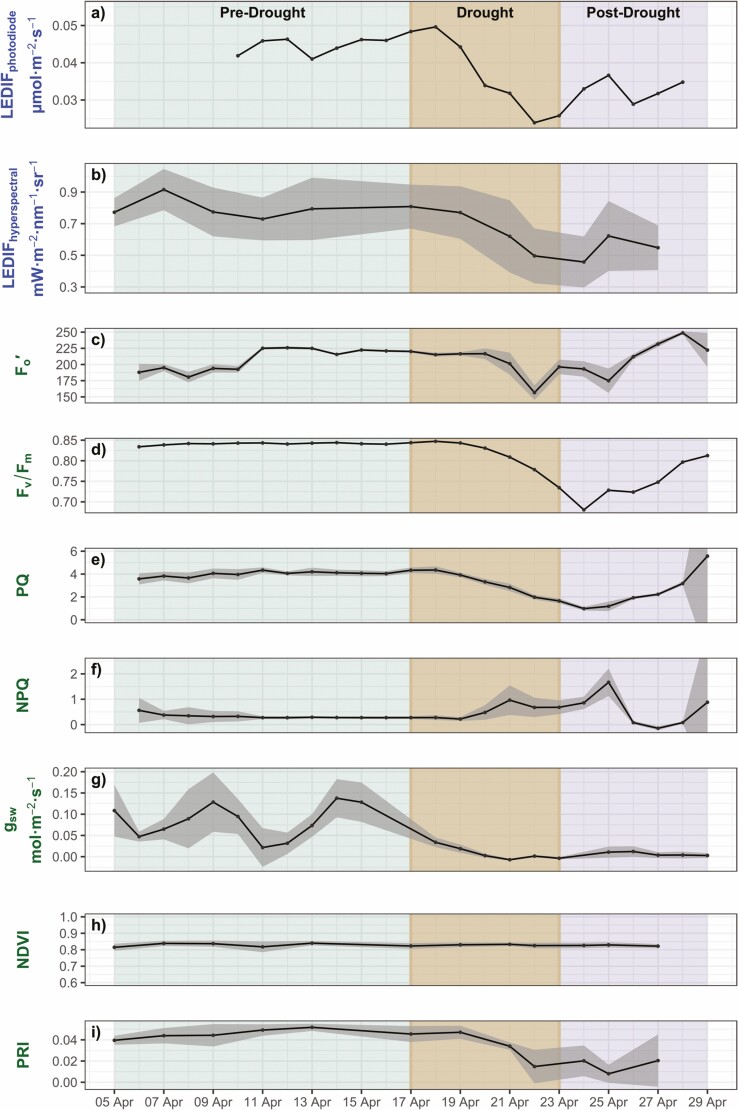
Distinct periods throughout the study are indicated by distinct colours: green (5 Apr - 17 Apr) indicates pre-drought, brown (17 Apr - 23 Apr) indicates experimentally imposed drought, and purple (23 Apr - 29 Apr) indicates post-drought. The top two rows (a,b) indicate canopy-level measurements, subplots d-i are leaf-level measurements. Note that not all measurements have the same *y*-axis scale. Panel A shows the continuous low-cost broadband photodiode sensor LEDIF data and Panel B shows the hyperspectral spectroradiometer LEDIF data. Panels C–F show time-series of leaf-level parameters collected from the MONI-PAM instrument. Panel G shows the stomatal conductance at leaf level from the LI-600 porometer. Panels H–I show time-series of calculated leaf-level vegetation indices (NDVI and PRI). For canopy-level: shaded regions indicate the standard deviation between the four scans taken on each sampling day. For leaf-level: shaded regions indicate the standard deviation between the 12 leaves scanned on each sampling day.

A typical drought response was observed across all parameters during the 25-day experiment (Fig. 3). There was a noticeable 1–2-day lag in response to the start of drought and return of normal watering conditions. At the canopy-level both LED fluorescence metrics, LEDIF_photodiode_ and LEDIF_hyperspectral_, decreased markedly when drought was induced (Fig. 3A and B). The relative decrease in LEDIF_photodiode_ was −50 % during this time period, while the change in LEDIF_hyperspectral_ was −44 %, indicating that the change in magnitude was similar. At the leaf level, NPQ increased whereas stomatal conductance (*g*_sw_­), *F*_v_/*F*_m_, PQ and ${F_{o}}^{\prime }$ decreased (Fig. 3C–G). In the short post-drought period, most metrics started to return to their baseline values as established in the pre-drought period. Notably, *g*_sw_ never returned to ‘pre-drought’ values. Leaf-level NDVI had little to no change over the entire duration of the experiment and never dropped below 0.8 (Fig. 3H). On the other hand, leaf-level PRI had a clear response to drought, mimicking the trends found in other leaf-level metrics. There was a dramatic decline in PRI ~3 days after the watering ceased and a slight increase ~1 day after watering was reinitiated (Fig. 3I).

**Figure 4.**
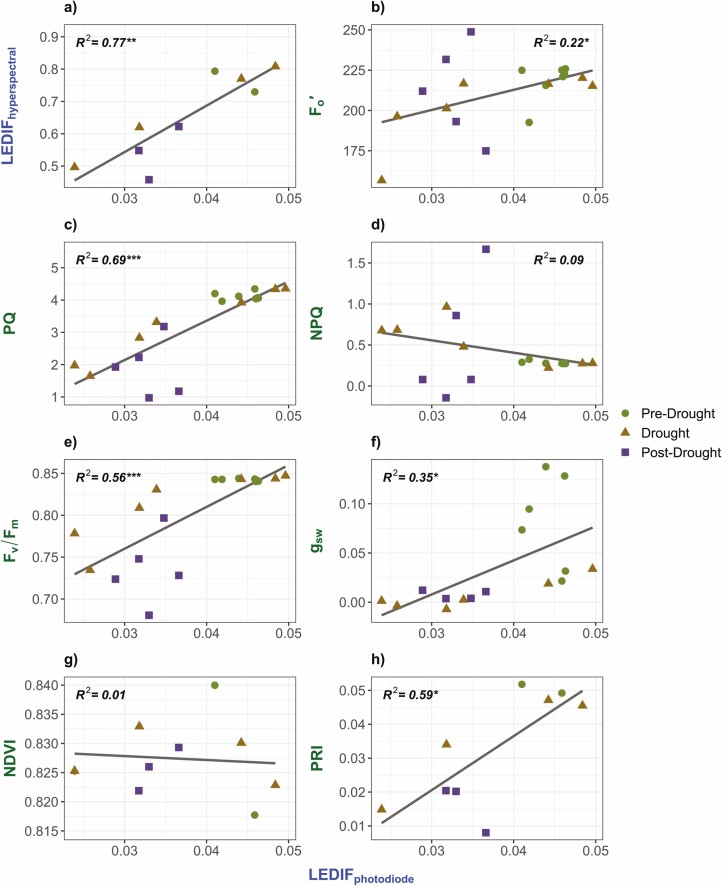
Correlations between canopy-level LEDIF_photodiode_ and (A) LEDIF_hyperspectral_, leaf-level (B)${F_{o}}^{\prime }$, (C) PQ, (D) NPQ, (E) *F*_v_/*F*_m_, (F) stomatal conductance, (G) NDVI and (H) PRI. Blue text refers to canopy-level measurements, green text refers to leaf-level measurements. Shapes refer to watering periods: green circle = pre-drought, brown triangle = drought, purple square = post-drought. The correlation and line fits are for all data points collected throughout the experiment; the stars represent *P* values: *<0.05, **<0.01, ***<0.001.

Linear regressions were performed to compare LEDIF_photodiode_ values with the spectrometer measurements. At the canopy-level, LEDIF_hyperspectral_ significantly correlated with LEDIF_photodiode_ (*R*^2^ = 0.77, *P* < 0.01) (Fig. 4A). Further, canopy-level LEDIF_photodiode_ had a weaker relationship, with leaf-level steady-state fluorescence (${F_{o}}^{\prime }$), measured with the MONI-PAM (*R*^2^ = 0.22, *P* < 0.05) (Fig. 4B). Other leaf-level PAM collected metrics also correlated with LEDIF_photodiode_, with varying levels of significance and *R*^2^ values (Fig. 4C–E). Stomatal conductance also correlates with steady-state canopy fluorescence—(*R*^2^ = 0.35, *P* < 0.05)—(Fig. 4F). As for the VIs, we did not find any significant trend between LEDIF_photodiode_ and NDVI, but there was a positive trend (*R*^2^ = 0.59, *P* < 0.05) between PRI and LEDIF_photodiode_ (Fig. 4G and H). Notably, most of the variance found in the regressions was found in the post-drought period data, except for *g*_sw_, where the variance was primarily found in the pre-drought period.

## DISCUSSION

In this study, we highlight changes in the drought response of canopy chlorophyll fluorescence in a broadleaf evergreen shrub using a novel low-cost night-time LED platform. Over the course of one month, we sought to meet two main objectives (1) track changes in canopy-level chlorophyll fluorescence with a new, night-time, low-cost sensor during an imposed stress event and (2) test whether these changes are reflected at the canopy and leaf level. By incorporating the use of LEDs to induce low-light steady-state chlorophyll fluorescence (Atherton *et al.* 2019) and dovetailing the inexpensive broadband photodiode sensors into our novel platform, we show that a low-cost LED/photodiode platform can be used to track dynamics in canopy fluorescence emission.

### Physiological responses

Stomatal conductance, g_sw_, which can be related to plant stress and water status (Buckley 2019) was measured throughout the experiment (Table 1). During the drought, we saw stomatal conductance quickly decrease (Fig. 3G), signalling a closure of the stomata. Stomatal closure is a predictable reaction of the plant to stop water loss when water is limited (Buckley 2019). When stomata close, carbon uptake becomes restricted (leaf gas exchange slows) and, therefore, a reduction in photosynthesis (Ball *et al.* 1987). Although changes in the stomata and photochemistry are not always necessarily coordinated (Marrs *et al.* 2020), we did find evidence of some coordination in our data, where *g*_sw_ was declining prior to PQ or NPQ decreasing and increasing, respectively (Fig. 3G, E and F). This suggests that the stomata were responding more quickly than the photochemical reactions, which has been observed at both the leaf- and canopy-level (Flexas *et al.* 2002; Magney Magney *et al.* 2020; Marrs *et al.* 2020). Notably, even after the drought ended, stomatal activity did not recover to pre-drought values. In a vineyard drought experiment, Tombesi *et al.* (2015) similarly found that even after resuming watering post-drought, stomata in the grape leaves did not reopen. The fact that stomatal conductance did not trend towards recovery, is notable in comparison to all other parameters measured, which increased post-drought (Fig. 3). Additionally, in a drought stress experiment of kidney bean plants where the drought lasted 7+ days, Miyashita *et al.* (2005) found that stomatal conductance was unable to fully recover post-drought, however, photochemistry recovered to more than 75 % of its pre-drought levels. Furthermore, after a prolonged drought period, stomatal conductance was the only parameter, when regressed against LEDIF_photodiode_, where most of the variance was held in the pre-drought data versus the post-drought data (Fig. 4F). This might suggest increased photorespiration in the droughted plants, and further decoupling between the photochemical reactions and stomatal activity (Wingler *et al.* 1999).

With reduced leaf-gas exchange, as suggested from our stomatal conductance measurements, we consequently saw a decrease in photochemical activity and altered energy balance, as shown through our PAM parameters (Fig. 3C–F). An increase in NPQ, as seen in our experiment, is a protective process in which excess light energy is dissipated as heat, which can also protect photosystem II from photo-oxidative damage (Baker 2008). Concurrently, PQ would be expected to decrease due to the lack of internal CO_2_ and overall saturation of photosynthesis by light causing the subsequent reduction in active PSII reaction centres (Maxwell and Johnson 2000; Baker 2008). As expected, during the drought, we saw an increase in NPQ, a subsequent decrease in PQ, as well as a decrease in *F*_v_/*F*_m_ and ${F_{o}}^{\prime }$ (Fig. 3F-c, respectively). Additionally, *F*_v_/*F*_m_—an indicator of plant photosynthetic performance (Maxwell and Johnson 2000)—decreased dramatically during the drought, indicating an uptick sustained NPQ (Maxwell and Johnson 2000), further detailing the physiological stress that the plant was experiencing.

We additionally used remotely sensed proxies of light absorption and photoprotective pigments (chlorophylls and carotenoids/xanthophylls) during our induced drought. PRI provides insight into photoprotective pigments—carotenoids that dynamically convert to expel excess absorbed energy when the plant is under stress or photosynthesis is saturated and can, therefore, provide us a proxy of photosynthetic efficiency (Gamon *et al.* 1992, 1997; Demmig-Adams and Adams 1996). During the drought period, we observed a reduction in PRI that directly mimicked the physiological parameters that were directly measuring the photosynthetic efficiency and PQ (Fig. 3I). Although Gamon *et al.* (1992) found that PRI did not perform as well in water-stressed sunflowers at the canopy level, our leaf-level PRI data did provide insight into photosynthetic activity compared to PAM parameters and spectral canopy measurements. When PRI values were regressed against LEDIF_photodiode_, we saw a relatively strong correlation, *R*^2^ = 0.59, *P* < 0.05 (Fig. 4H), with a sharp decrease in the drought period and inklings of recovery in the post-drought period, as would be expected (Magney *et al.* 2016; Wong *et al.* 2022). We also compared results against NDVI, which provides insight into the ‘greenness’ of the plant and its chlorophyll content. NDVI did not prove insightful in detecting plant stress during the week-long imposed drought as there was little to no change in NDVI throughout the experiment (Fig. 3H), and there was no visible change in greenness throughout the experiment. This makes intuitive sense given the short duration of the experiment—suggesting there was likely no change in leaf structure or chlorophyll concentration, making NDVI invariant.

When comparing the photodiode and hyperspectral LEDIF retrieval methods, our results demonstrated similar performance (Figs 3 and 4; *R*^2^ = 0.77, *P* < 0.01). This shows promise for a low-cost method to track temporal changes in canopy fluorescence emission (Fig. 3A and B; Fig. 4A). Not only this, but LEDIF_photodiode_ tracked similar patterns in drought response of photosynthetic status indicated by PAM fluorescence measurements (Fig. 4). LEDIF_photodiode_ correlated well with *F*_*v*_/*F*_*m*_ (*R*^2^ = 0.59, *P* < 0.001) indicating that we were able to capture changes in photosynthetic capacity and fluctuations of NPQ. Furthermore, LEDIFphotodiode also correlated with PQ (*R*^2^ = 0.69, *P* < 0.001) indicating that we were also able to track the leaf-level capacity of PSII at the canopy-level. With this, LEDIF_photodiode_ indeed seems to be a meaningful indicator of photosynthetic status, and thus, overall plant status.

### Future considerations

While the results of this experiment demonstrate the potential for using night-time LEDIF_photodiode_ measurements to track canopy-level chlorophyll fluorescence in a cost-effective way, there are some limitations. Although we detected a fluorescence emission (Fig. 2), instigated by the blue LEDs, the measured radiance was relatively small (Fig. 2A). This may limit the method’s effectiveness when implemented in larger canopies or if the platform is placed higher above the canopy. Future studies may need to use stronger (greater than 15 W) LEDs to overcome the weak signal. However, we caution that too strong of an LED intensity can have unintended consequences on plant physiology, for example, stomatal opening; ideally, increasing the signal-to-noise ratio while not inducing a photosynthetic response would be preferred. In this sense, an increase in detector sensitivity (i.e. choice of photodiode sensor) is desired, rather than increasing the incidence illumination. Additionally, if users seek to validate the LEDIF_photodiode_ approach with hyperspectral measurements as was done here, they might consider a longer integration time than 1s, as this will help to smooth out the spectral shape of Chl *a* fluorescence (Fig. 2B). In addition, further research is needed to determine the effectiveness of this method in uncontrolled settings, such as croplands or forests *in situ*, and at distances greater than 0.5 m from the top of the canopy. Ultimately, this method may serve as a way to validate SIF-yield data collected from tower-level measurements and species-specific responses within the field of view of a tower.

Strong stress may also induce structural changes in the canopy (e.g. wilting). It is challenging to separate structural changes from physiological changes when using remote sensing methods to measure photosynthesis or, in our case, chlorophyll fluorescence. Even though a structural change was not observed in our study, understanding how to disentangle or account for structural changes when measuring canopy fluorescence could potentially improve the interpretation and generalizability of the results. One approach may be to normalize the far-red LEDIF by reflected light in the LED light region (e.g. blue light). This could help account for variations in canopy structure to enhance the SIF signal (Magney *et al.* 2019; Pierrat *et al.* 2021).

In summary, the successful implementation of our labour-reducing, low-cost canopy-based, night-time LEDIF system in this study demonstrates its potential for use in future research and highlights the need for continued exploration of the capabilities and limitations of such remote sensing tools. The development of new and innovative tools to measure abiotic plant stress is crucial for advancing our understanding of plant physiological responses to environmental stressors, the connections between the physiological mechanisms linking chlorophyll fluorescence and photosynthesis at multiple scales, and for the development of effective strategies for mitigating the impacts of climate change in our natural ecosystems.

## Data Availability

Data are available at Brissette and Magney (2023).
